# Endothelin-1 Augments Na^+^/H^+^ Exchange Activity in Murine Pulmonary Arterial Smooth Muscle Cells via Rho Kinase

**DOI:** 10.1371/journal.pone.0046303

**Published:** 2012-09-28

**Authors:** Clark Undem, Eon J. Rios, Julie Maylor, Larissa A. Shimoda

**Affiliations:** Division of Pulmonary and Critical Care Medicine, Department of Medicine, Johns Hopkins School of Medicine, Baltimore, Maryland, United States of America; University of Giessen Lung Center, Germany

## Abstract

Excessive production of endothelin-1 (ET-1), a potent vasoconstrictor, occurs with several forms of pulmonary hypertension. In addition to modulating vasomotor tone, ET-1 can potentiate pulmonary arterial smooth muscle cell (PASMC) growth and migration, both of which contribute to the vascular remodeling that occurs during the development of pulmonary hypertension. It is well established that changes in cell proliferation and migration in PASMCs are associated with alkalinization of intracellular pH (pH_i_), typically due to activation of Na^+^/H^+^ exchange (NHE). In the systemic vasculature, ET-1 increases pH_i_, Na^+^/H^+^ exchange activity and stimulates cell growth via a mechanism dependent on protein kinase C (PKC). These results, coupled with data describing elevated levels of ET-1 in hypertensive animals/humans, suggest that ET-1 may play an important role in modulating pH_i_ and smooth muscle growth in the lung; however, the effect of ET-1 on basal pH_i_ and NHE activity has yet to be examined in PASMCs. Thus, we used fluorescent microscopy in transiently (3–5 days) cultured rat PASMCs and the pH-sensitive dye, BCECF-AM, to measure changes in basal pH_i_ and NHE activity induced by increasing concentrations of ET-1 (10^−10^ to 10^−8^ M). We found that application of exogenous ET-1 increased pH_i_ and NHE activity in PASMCs and that the ET-1-induced augmentation of NHE was prevented in PASMCs pretreated with an inhibitor of Rho kinase, but not inhibitors of PKC. Moreover, direct activation of PKC had no effect on pH_i_ or NHE activity in PASMCs. Our results indicate that ET-1 can modulate pH homeostasis in PASMCs via a signaling pathway that includes Rho kinase and that, in contrast to systemic vascular smooth muscle, activation of PKC does not appear to be an important regulator of PASMC pH_i_.

## Introduction

Pulmonary hypertension can result from a variety of etiologies, including genetic mutations, environmental factors (i.e, anorexigens), and hypoxia due to chronic lung diseases [1]–[3]. In all cases, increased muscularization of the vasculature and enhanced vasomotor tone contribute to the elevation in pulmonary arterial pressure. The exact mechanisms underlying the pathogenesis of PASMC growth are not known, but studies have shown that increased intracellular pH (pH_i_) accompanies cell proliferation in systemic [4]–[6] and pulmonary [7] vascular smooth muscle cells.

In mammalian cells, pH_i_ homeostasis is maintained in large part by several membrane bound transporters, including the Na^+^-HCO_3_^-^ co-transporter, Na^+^-dependent Cl^−/^HCO_3_^-^ exchange, Na^+^-independent Cl^−/^HCO_3_^-^ exchange and Na^+^/H^+^ exchange, all of which have been shown to be functionally present and contribute to control of pH_i_ in vascular smooth muscle [8]–[12]. In PASMCs, the use of Na^+^/H^+^ exchanger (NHE) antagonists revealed that this transporter plays a significant role in regulating resting pH_i_ [11], [13]. Na^+^/H^+^ exchangers are plasma membrane spanning proteins that use the transmembrane Na^+^ gradient to extrude protons. Many stimuli, including acute [10] and chronic hypoxia [13] and growth factors [7] induce PASMC alkalinization. The increase in pH_i_ observed in response to chronic hypoxia or platelet-derived and epidermal growth factor was demonstrated to require activation of NHE activity [7], [13]. Moreover, inhibition of Na^+^/H^+^ exchange with amiloride analogs or selective knockdown of NHE isoform 1 (NHE1) prevents PASMC proliferation in response to growth factors and attenuated vascular remodeling and pulmonary hypertension in rodents exposed to chronic hypoxia, respectively [7], [14], [15]. The results from these studies indicate that enhanced NHE activity in response to growth factors is an important component in modulating pH_i_ and PASMC growth.

Since its discovery in 1988 [16], ET-1 has emerged as a strong candidate in mediating the development and progression of pulmonary hypertension. ET-1 is one of the most potent and abundant endothelial-derived constricting factors identified to date, and has mitogenic and anti-apoptotic properties [17]–[21]. Three isoforms of endothelin (ET-1, ET-2, ET-3) have been identified, of which ET-1 is the most widely distributed, and thus, the most widely studied. ET-1 was initially identified as a secretory product from aortic endothelial cells [16] and is primarily produced in, and secreted from, vascular endothelium. ET-1 levels are markedly increased in almost all forms of pulmonary hypertension, and ET-1 receptor antagonists prevent and partially reverse the development of hypoxic pulmonary hypertension in animal models [22]–[30] and are now used clinically in the management of many forms of pulmonary hypertension [31]. While ET-1 was shown to increase pH_i_, NHE activity and cell growth in systemic vascular smooth muscle [32], the effects of ET-1 on PASMC pH homeostasis are unknown.

Two endothelin receptor subtypes have been identified and characterized: ET_A_ and ET_B,_ both of which mediate proliferation in PASMCs [33]. Once ET-1 binds to its surface receptor, a complex signaling process is set in motion. In general, endothelin receptors are G-protein coupled to the phospholipase C cascade, leading to increased [Ca^2+^]_i_ and activation of protein kinase C (PKC) and Rho kinase (ROCK) [34]–[37]. That PKC activation leads to increased NHE activity has been well documented in systemic vascular smooth muscle [38]–[41], and in several cell types the action of ET-1 on Na^+^/H^+^ exchange was confirmed to require PKC [42]–[44].

Despite the fact that overwhelming evidence suggests that alterations in pH_i_ and Na^+^/H^+^ exchange are necessary for vascular smooth muscle cell growth, and that ET-1 levels are elevated pulmonary hypertension patients and animal models of pulmonary hypertension, the effect of ET-1 on basal pH_i_ and NHE activity has yet to be examined in PASMCs. Thus, in this study, we used fluorescent microscopy to measure basal pH_i_ and NHE activity to test the hypothesis that challenge with ET-1 would lead to PKC-dependent activation of NHE activity and a consequent alkaline shift in pH_i_ in PASMCs.

## Methods

### Ethics Statement

All procedures were performed in strict accordance with the recommendations in the Guide for the Care and Use of Laboratory Animals of the National Institutes of Health and were approved by the Animal Care and Use Committee of The Johns Hopkins University School of Medicine (Protocol number MO06M161).

### Cell Isolation and Culture

**Figure 1:**
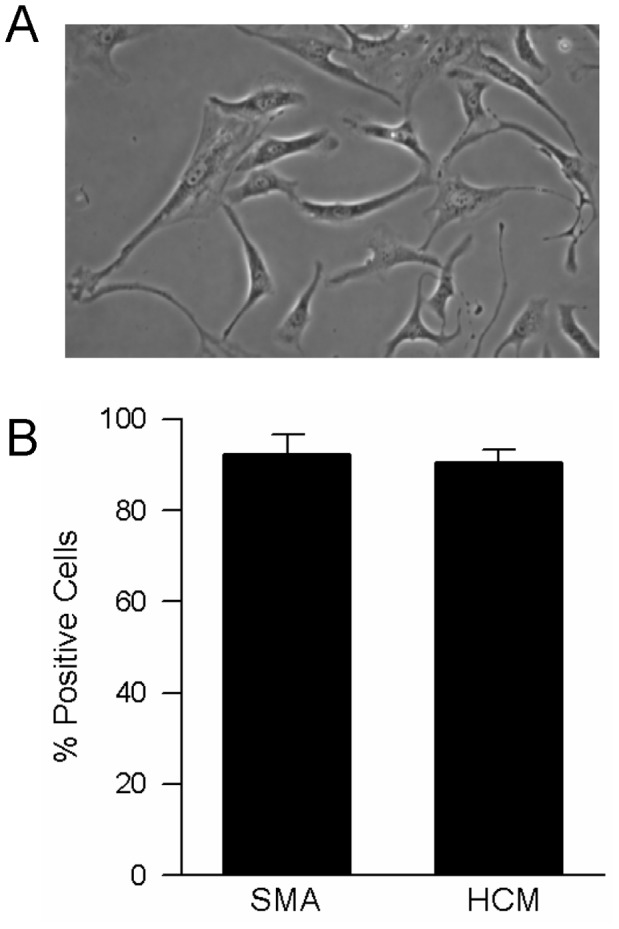
Characterization of murine pulmonary arterial smooth muscle cells (PASMCs). **A)** Representative phase contrast image showing morphology of murine PASMCs. **B)** Bar graph showing mean±SEM values (n = 3-4 isolations) for the percent of cells staining positive for the smooth muscle cell markers smooth muscle specific α-actin (SMA) and heavy chain myosin (HCM).

The method for obtaining single PASMCs has been described previously [13]. Briefly, adult, male C57/B6 mice were anesthetized with sodium pentobarbital (65 mg/kg i.p.), and under deep anesthesia the heart and lungs removed and transferred to a petri dish containing HEPES-buffered salt solution (HBSS) containing (in mmol/L): 130 NaCl, 5 KCl, 1.2 MgCl_2_, 1.5 CaCl_2_, 10 N-[2-hydroxyethyl]piperazine-N’-[2-ethanesulfonic acid] (HEPES) and 10 glucose, with pH adjusted to 7.2 with 5 mol/L NaOH. Intrapulmonary arteries (100–400 µm outer diameter) were isolated and cleaned of connective tissue. After disrupting the endothelium by gently rubbing the luminal surface with a cotton swab, the arteries were allowed to recover for 30 min in cold (4°C) HBSS, followed by 20 min in reduced-Ca^2+^ (20 µmol/L CaCl_2_) HBSS at room temperature. The tissue was digested in reduced-Ca^2+^ HBSS containing collagenase (type I; 1750 U/ml), papain (9.5 U/ml), bovine serum albumin (2 mg/ml) and dithiothreitol (1 mmol/L) at 37°C for 10 minutes. Following digestion, single smooth muscle cells were dispersed by gentle trituration with a wide-bore transfer pipette in Ca^2+^-free HBSS and the cell suspension was placed on 25 mm glass cover slips. PASMCs were cultured under normoxic conditions in SmBm complete media (Lonza) supplemented with 10% fetal calf serum for 2–4 days and placed in serum-free media 24 hr before experiments. The identity of the cells under study was confirmed as smooth muscle by elongated, spindle morphology (Fig 1A), and positive staining for both smooth muscle specific α-actin (SMA) and heavy chain myosin (HCM) (Fig 1B). For immunofluorescence, cells were grown on glass coverslips, fixed with 10% formalin, washed with PBS, permeabilized with 0.5% Triton-X and blocked with 20% goat serum. Cells were then incubated with monoclonal antibodies against SMA (Sigma-Aldrich) or HCM (Abcam), followed by fluorescent secondary antibody (CY3; Molecular Probes) and the nuclear dye, YO-PRO. Random fields were examined to achieve a minimum total cell count of 100 per animal, and the number of cells exhibiting SMA/HCM positivity calculated as a percent of total cell number.

### Intracellular pH Measurements

PASMCs were placed in a laminar flow cell chamber perfused with HBSS with pH adjusted to 7.4. pH_i_ was measured in cells incubated with the membrane permeant (acetoxymethyl ester) form of the pH-sensitive fluorescent dye 2′,7′-bis(carboxyethyl)-5(6)-carboxyfluorescein (BCECF-AM) for 60 min at 37°C under an atmosphere of 20% O_2_-5% CO_2_. Cells were then washed with HBSS for 15 min at 37°C to remove extracellular dye and allow complete de-esterification of cytosolic dye. Ratiometric measurement of BCECF fluorescence was performed on a workstation (Intracellular Imaging Inc, Cincinnati, OH) consisting of a Nikon TSE 100 Ellipse inverted microscope with epi-fluorescence attachments. The light beam from a xenon arc lamp was filtered by interference filters at 490 and 440 nm, and focused onto the PASMCS under examination via a 20× fluorescence objective (Super Fluor 20, Nikon). Light emitted from the cell at 530 nm was returned through the objective and detected by an imaging camera. An electronic shutter (Sutter Instruments) was used to minimize photobleaching of dye. Protocols were executed and data collected on-line with InCyte software (Intracellular Imaging Inc). pH_i_ was estimated from *in situ* calibration after each experiment. Cells were perfused with a solution containing (in mmol/L): 105 KCl, 1 MgCl_2_, 1.5 CaCl_2_, 10 glucose, 20 HEPES-Tris and 0.01 nigericin to allow pH_i_ to equilibrate to external pH. A two point calibration was created from fluorescence measured as pH_i_ was adjusted with KOH from 6.5 to 7.5. Intracellular H^+^ ion concentration ([H^+^]_i_) was determined from pH_i_ using the formula: pH_i_  =  −log ([H^+^]_i_).

### Experimental Protocols

#### Effect of ET-1and PMA on basal pH_i_

Baseline pH_i_ was measured for five minutes in PASMCs under control conditions. Values were averaged to obtain a mean value for each cell. The effect of agonists on baseline pH_i_ was then determined by monitoring pH_i_ for an additional 10 min in the same cells during exposure to ET-1 (10^−10^ to 10^−8^ M) or phorbol 12-myristate 13-acetate (PMA; 1 µmol/L). Each concentration was tested on a separate coverslip of cells.

#### Effect of antagonists on basal pH_i_

The effect of antagonists on basal pH_i_ was determined in HBSS-perfused cells. After obtaining a stable baseline for 5 min, cells were then perfused with antagonists for 10 min. For Y-27632, cells were pretreated for 30 min to allow complete cell penetration and ROCK inhibition. Thus, the effect of Y-27632 on basal pH_i_ was determined by comparing baseline in paired coverslips (control and Y-27632-treated). To determine the effect of antagonists on the ET-1-induced increase in pH_i_, cells were then challenged for 10 min with 10^−8^ mol/L ET-1 in the presence of antagonist.

#### Effect of ET-1on Na^+^/H^+^ exchange

**Figure 2:**
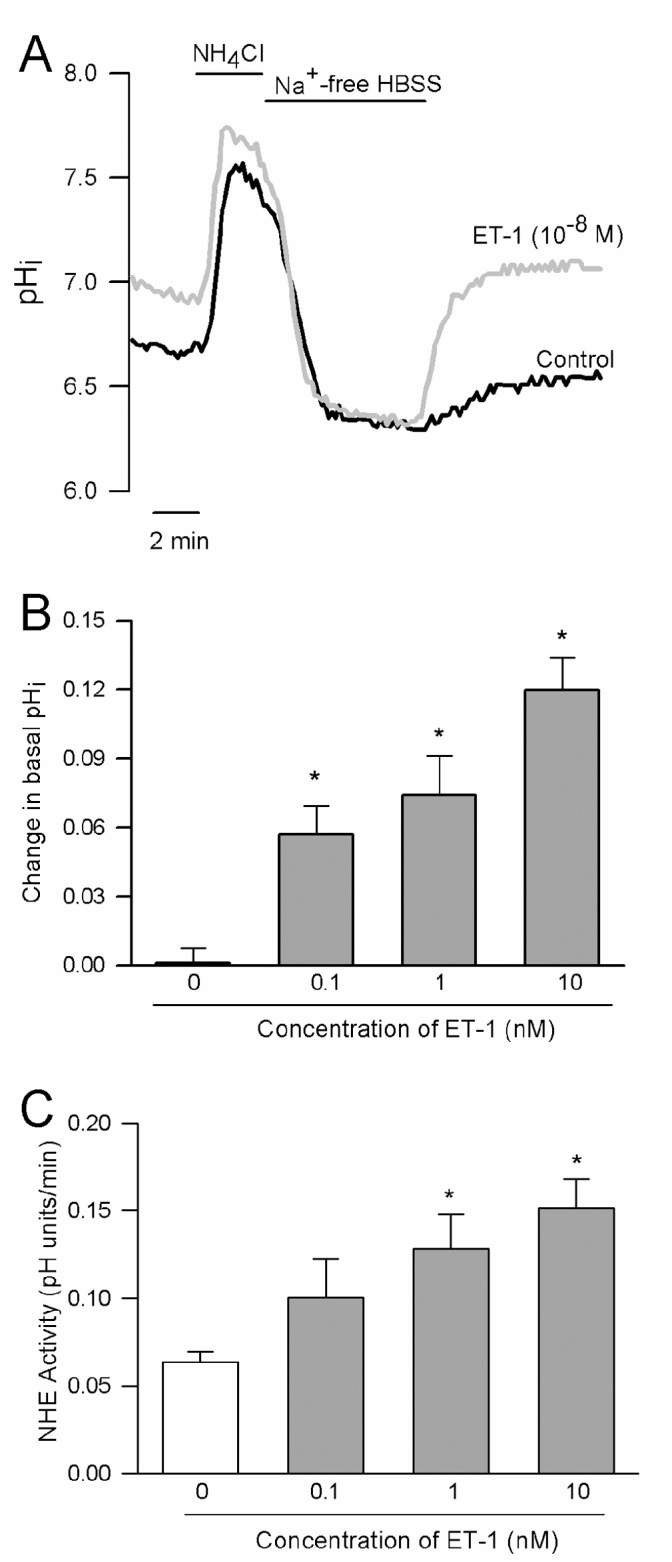
Effect of endothelin-1 (ET-1) on intracellular pH (pH_i_) and Na^+^/H^+^ exchanger (NHE) activity. **A)** Representative traces showing pH_i_ measured during the ammonium pulse protocol in control cells and cells treated with ET-1 (10^-8^ M). **B and C)** Bar graphs showing mean±SEM values for **B)** the change (Δ) in baseline pH_i_ (n = 3-6) and **C)** NHE activity (n = 5-15) induced by different concentrations of ET-1. * indicates significant difference from control (0 nmol/L ET-1)

A standard ammonia pulse technique was used to measure NHE activity (Figure 2A). PASMCs loaded with BCECF were perfused at a rate of 1 mL/min with Solution 1 containing (in mmol/L): 130 NaCl, 5 KCl, 1MgCl_2_, 1.5 CaCl_2_, 10 glucose and 20 HEPES with pH adjusted to 7.4 with NaOH at 37°C. Baseline pH_i_ was measured for 2 min before cells were briefly exposed to NH_4_Cl (ammonium pulse) by perfusing with Solution 2 containing (in mmol/L): 110 NaCl, 20 NH_4_Cl, 5 KCl, 1MgCl_2_, 1.5 CaCl_2_, 10 glucose, 20 HEPES at a pH of 7.4 using NaOH for 3 min. The ammonium pulse caused alkalinization due to influx of NH_3_ and buffering of intracellular H^+^ (Fig 2A). Washout of NH_4_Cl in the absence of extracellular Na^+^ using a Na^+^- and NH_4_^+^- free solution containing (in mmol/L): 130 choline chloride, 5 KCl, 1MgCl_2_, 1.5 CaCl_2_, 10 glucose and 20 HEPES at a pH of 7.4 using KOH for 10 min results in acidification due to rapid diffusion and washout of NH_3_. The external solution was then switched back to Na^+^-containing Solution 1 for 10 min. Re-addition of extracellular Na^+^ allows activation of Na^+^/H^+^ exchange and recovery from acidification to basal levels. The rate of Na^+^-dependent recovery from intracellular acidification (change in pH over 2 min) corresponds to NHE activity.

### Drugs and Solutions

ET-1 was obtained from American Peptides (Sunnyvale, CA). Y-27632 and GF 102903X were obtained from Calbiochem (La Jolla, CA). All other reagents were obtained from Sigma Aldrich (St. Louis, MO). ET-1 (10^–5 ^mol/L in deionized H_2_O) was made up in a stock solution, divided into aliquots, and stored at –20°C until used. Stock solutions of Y-27632 (10 mmol/L in deionized H_2_O) and PMA (0.1 mmol/L in deionized H_2_O) were made, aliquotted, and stored at 0°C until used. GF 109203X (GFX; 10 mmol/L in DMSO), and staurosporine (Stauro; 10 mmol/L in DMSO) were made up in a stock solution and stored at 4°C. A stock solution of 5-(N,N-Dimethyl)amiloride hydrochloride (DMA; 10 mmol/L in deionized H_2_O) was made and used on the day of the experiment. All stock solutions were diluted to working concentrations in perfusate on the day of experiment.

### Data Analysis

All values are expressed as mean ± SEM. In each experiment, data was collected from up to 30 cells, and the values averaged to obtain a single value for each experiment. Cells isolated from different animals were used in each experiment, thus, “n” refers to both the number of experiments as well as number of animals from which cells were derived. Although presented as pH_i_ values in the text and figures, pH values were converted to [H^+^]_i_ prior to running statistics. Change in pH_i_ (ΔpH_i_) was computed by subtracting the average basal pH_i_, determined from 1 min of data collected immediately prior to beginning challenge, from the average of five data points at the peak of the response. For each agonist (ET-1 and PMA), all data were compared against a single control group as a single analysis using a one-way ANOVA with a Holm-Sidak *post hoc* test to determine differences between groups. In some cases, a one-sample t-test was used to determine whether the change in pH_i_ observed after treatment was statistically different from zero (i.e., value for the ΔpH_i_ is significantly different from the test value of 0). A P value <0.05 was accepted as statistically significant.

## Results

### Effect of ET-1 on pH_i_ and NHE Activity

When monitored over 10 min, basal pH_i_ was stable and no significant change in pH_i_ was observed. However, application of ET-1 caused concentration-dependent increases in pH_i_ (Fig 2B), with an average change of greater than 0.1 pH units at 10^−8^ mol/L ET-1, a concentration that we previously found caused maximal contraction in pulmonary arteries [45], [46]. The ammonium pulse technique was used to measure the effect of ET-1 on NHE activity (Fig 2A). PASMCs were exposed to different concentrations of ET-1 for 15 min before beginning the ammonium pulse. Consistent with the effects of ET-1 on baseline pH_i_, the rate of Na^+^-dependent recovery from acid-loading (NHE activity), measured during the first 2 min after re-addition of Na^+^, was greater in the presence of ET-1 (Fig 2C). Although increases in NHE activity were observed at all concentrations of ET-1 tested, with a greater than 2-fold increase in NHE activity at 10 nmol/L, the difference with 0.1 nmol/L ET-1 did not quite reach statistical significance.

**Figure 3:**
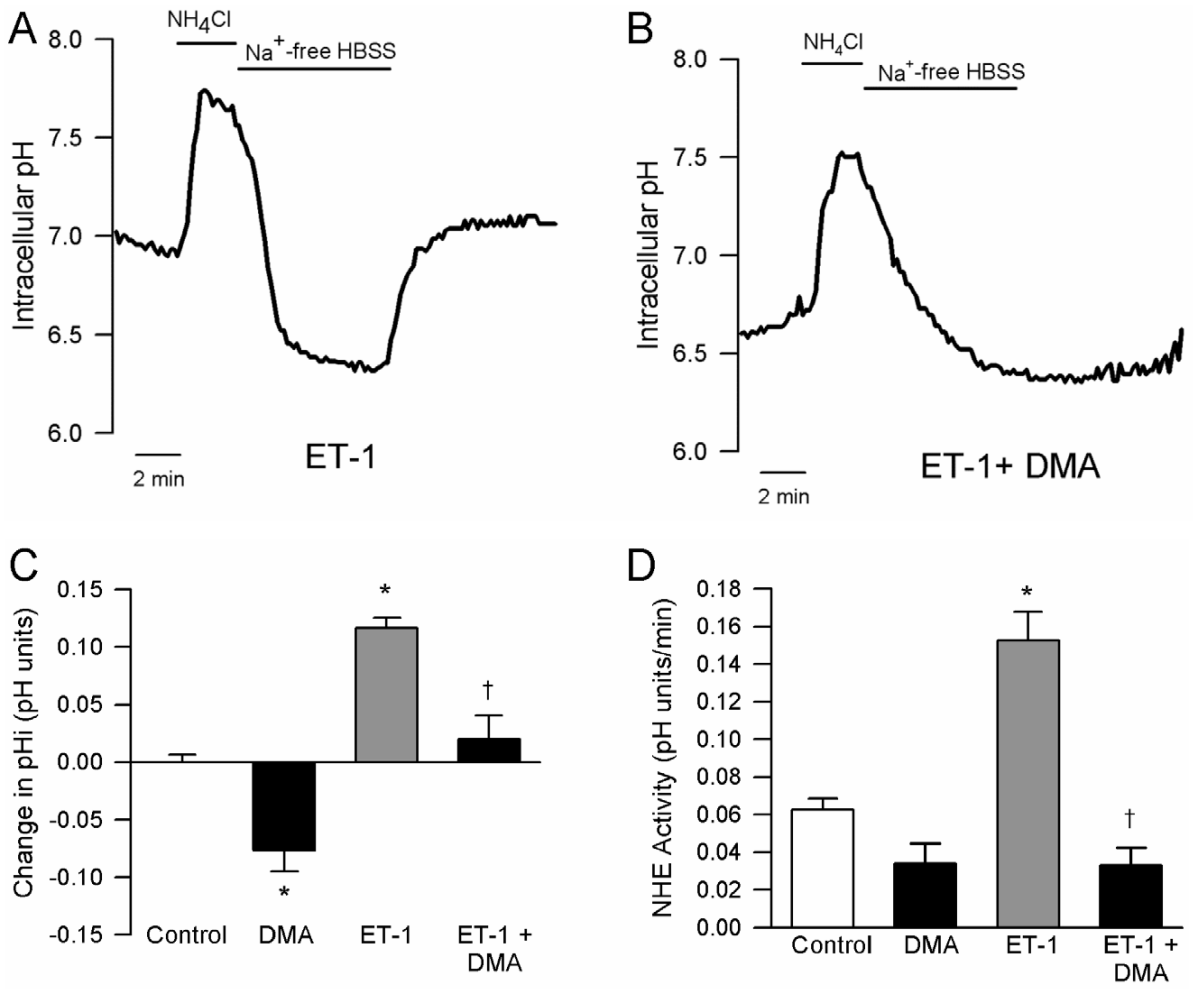
Effect of dimethyl amiloride (DMA; 1 μmol/L) on intracellular pH (pH_i_) and Na^+^/H^+^ exchanger (NHE) activity. **A and B)** Representative traces showing pH_i_ measured during the ammonium pulse protocol in cells treated with ET-1 (10^-8^ M) in the **A)** absence and **B)** presence of DMA. **C and D)** Bar graphs showing mean±SEM values for **C)** the change (Δ) in baseline pH_i_ (n = 3-6) and **D)** NHE activity (n = 5-15) in control (Con) cells or in cells treated with DMA alone (n =  4 for pH_i_ and n = 6 for NHE activity) , ET-1 (10^-8^ M; n =  5 for pH_i_ and n = 11 for NHE activity) and ET-1 + DMA (n = 6 for pH_i_ and n = 5 for NHE activity). * indicates significant difference from control; †indicates significant difference from ET-1 alone.

Although perfusion of cells with bicarbonate-free extracellular solution should eliminate contributions of the Cl^−/^HCO_3_^-^ exchangers to regulation of pH_i_, we verified that the increase in basal pH_i_ was due to increased NHE activity by repeating experiments in the presence of DMA (1 µmol/L), a NHE inhibitor (Fig 3A and B). Consistent with the known role of NHE in regulating pH_i_ in PASMCs, DMA caused a significant reduction in basal pH_i_ (Fig 3C). When applied in the presence of DMA, the ET-1-induced increase in basal pH_i_ was greatly reduced, with only a small, statistically insignificant increase in pH_i_ observed (Fig 3C). DMA also reduced NHE activity under control conditions (Fig 3D), although the reduction did not quite reach statistical significance (P = 0.06), and completely prevented the ET-1-induced increase in NHE activity.

### Role of PKC in Mediating the ET-1-induced Increase in NHE Activity

**Figure 4:**
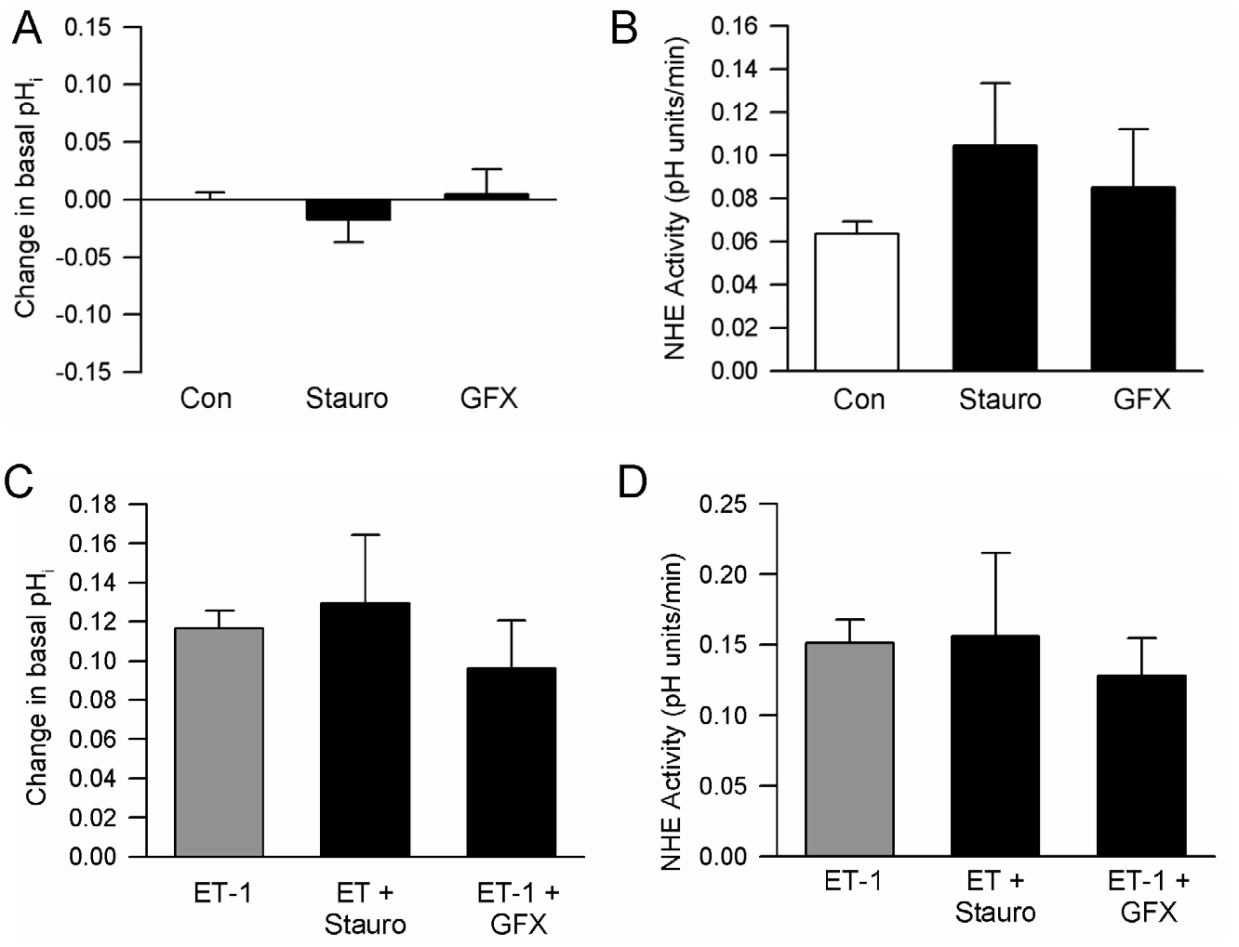
Effect of protein kinase C (PKC) inhibitors on intracellular pH (pH_i_) and Na^+^/H^+^ exchanger (NHE) activity. **A and B)** Change (Δ) in baseline **A**) pH_i_ and **B**) NHE activity induced by addition of staurosporine (Stauro; 30 nmol/L; n =  4 each) or GF109203X (GFX; 50 nmol/l; n = 4 each). **C** and **D**) Bar graphs show mean ±SEM for **C**) change in pH_i_ and **D**) NHE activity in response to ET-1 (10^-8^ mol/L) in the absence (n = 11) or presence of Stauro (n = 3) or GFX (n = 4).

PKC activation has been shown to enhance NHE activity in a variety of cell types, including vascular smooth muscle [38]–[41]. To determine whether the increase in PASMC pH_i_ and NHE activity in response to ET-1 was due to activation of PKC, cells were pretreated with two different PKC inhibitors: Stauro (50 nmol/L), a relatively nonselective PKC inhibitor, and GFX (30 nmol/L), a selective PKC inhibitor with greater affinity for Ca^2+^-dependent isoforms [47]. Neither inhibitor had a significant effect on basal pH_i_ (Fig 4A) or NHE activity (Fig 4B). Surprisingly, inhibiting PKC with either Stauro or GFX could not prevent the ET-1-induced increase in pH_i_ (Fig 4C) or NHE activity (Fig 4D).

### Effect of PMA on pH_i_ and NHE Activity

**Figure 5:**
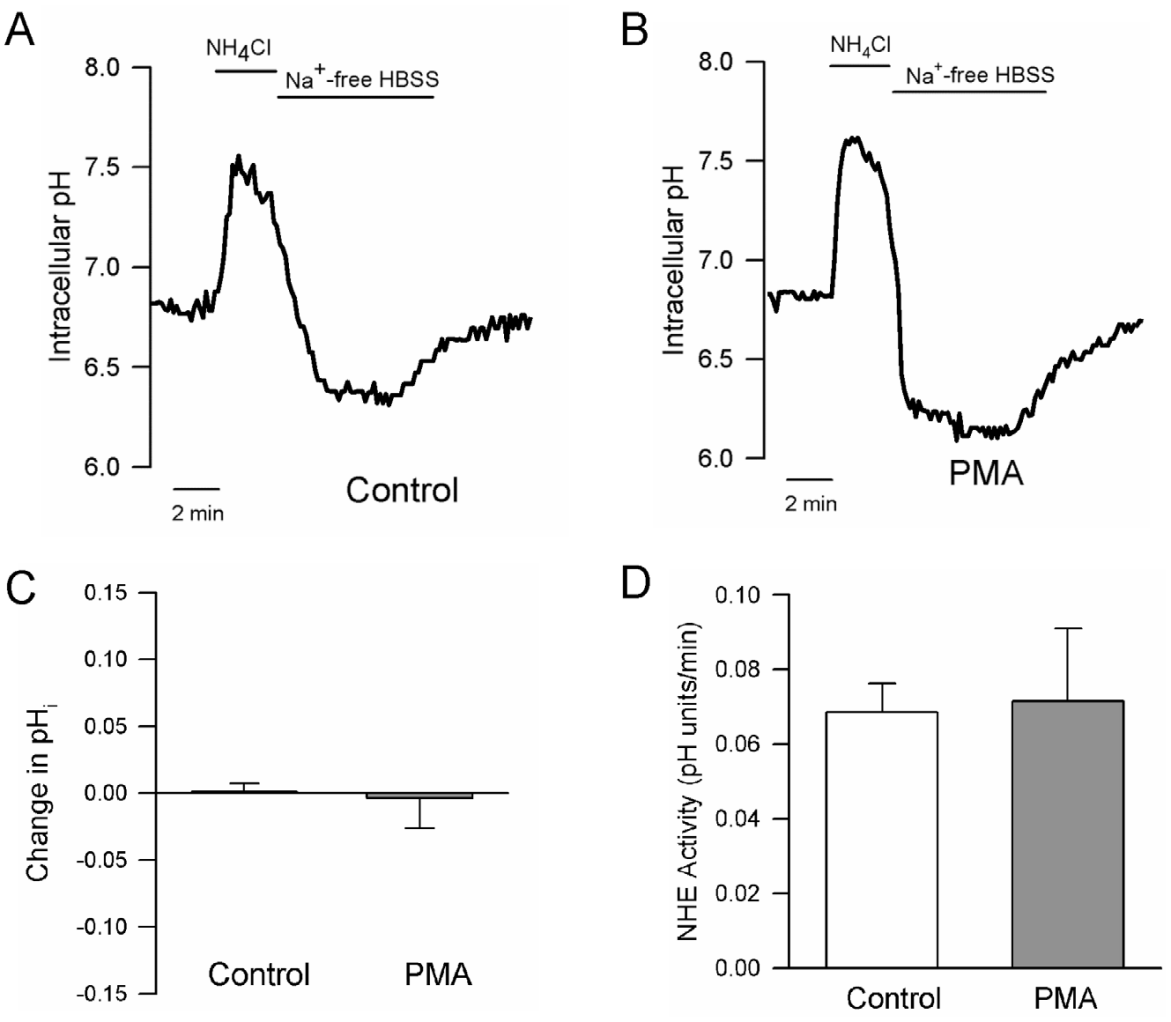
Effect of PKC activation on intracellular pH (pH_i_) and Na^+^/H^+^ exchanger (NHE) activity. Representative traces show pH_i_ measured during ammonium pulse in the **A)** absence and **B)** presence of PMA (1 μmol/L). **C and D)** Bar graphs show mean±SEM for **C)** the change (Δ) in baseline pH_i_ (n = 7) and **D)** NHE activity (n = 8) in response to PMA.

Since PKC inhibition failed to block ET-1-induced changes in pH_i_ and NHE activity, we tested whether PKC activation could stimulate NHE activity in PASMCs by challenging cells with PMA (500 nmol/L) (Fig 5A and B). Although we previously showed this concentration of PMA to be sufficient to induce Ca^2+^ influx and reduce K_V_ currents in PASMCs [37], [48], since this concentration of PMA appeared to have no observable effect on either baseline pH_i_ or NHE activity, we increased the concentration of PMA to 1 µmol/L. Perfusion with 1 µmol/L PMA for 15 min had no effect on basal pH_i_ (Fig 5C). Moreover, NHE activity was similar in control and PMA-treated cells (Fig 5D).

### Role of ROCK in Mediating ET-1-induced PKC Activation

**Figure 6:**
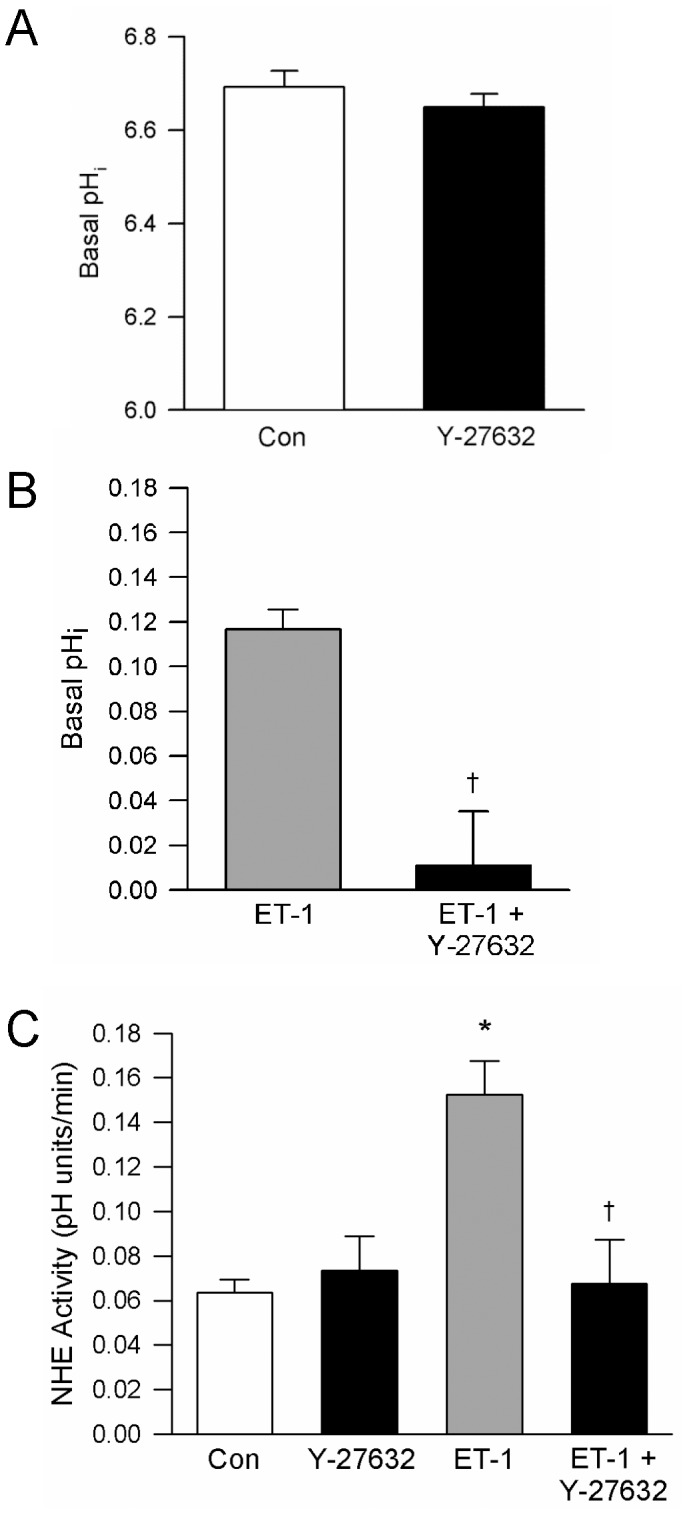
Effect of ROCK inhibition on intracellular pH (pH_i_) and Na^+^/H^+^ exchanger (NHE) activity. **A)** Bar graph showing mean±SEM values for basal pH_i_ measured in cells in the absence (n = 4) or presence (n = 4) of Y-27632 (10 μmol/L; 30 min). **B)** Bar graph shows mean±SEM values for the change (Δ) in baseline pH_i_ in response to ET-1 (10^-8^ mol/L) in the absence (n = 5) and presence (n = 3) of Y-27632. **C)** Bar graph showing mean±SEM for NHE activity in cells challenged with ET-1 in the absence (n = 11) and presence (n = 5) of Y-27632. * indicates significant difference from control; †indicates significant difference from ET-1 alone.

Since our results appeared to rule out a role for PKC in mediating the ET-1-induced activation of NHE activity, we next tested whether ROCK was involved using the ROCK inhibitor, Y-27632 (10 µmol/L). In order to allow sufficient time for Y-273632 to enter the cells, cells were pretreated with Y-27632 for 30 min prior to beginning experiments. Incubation with Y-27632 had no effect on basal pH_i_ (Fig 6A). When cells were challenged with ET-1 (10^−8^ mol/L) in the presence of Y-27632, no significant increase in pH_i_ was observed (Fig 6B). Consistent with the lack of effect of Y-27632 on basal pH_i_, ROCK inhibition did not alter NHE activity (Fig 6C); however, pretreatment with Y-27632 completely prevented the ET-1-induced increase in NHE activity.

## Discussion

**Figure 7:**
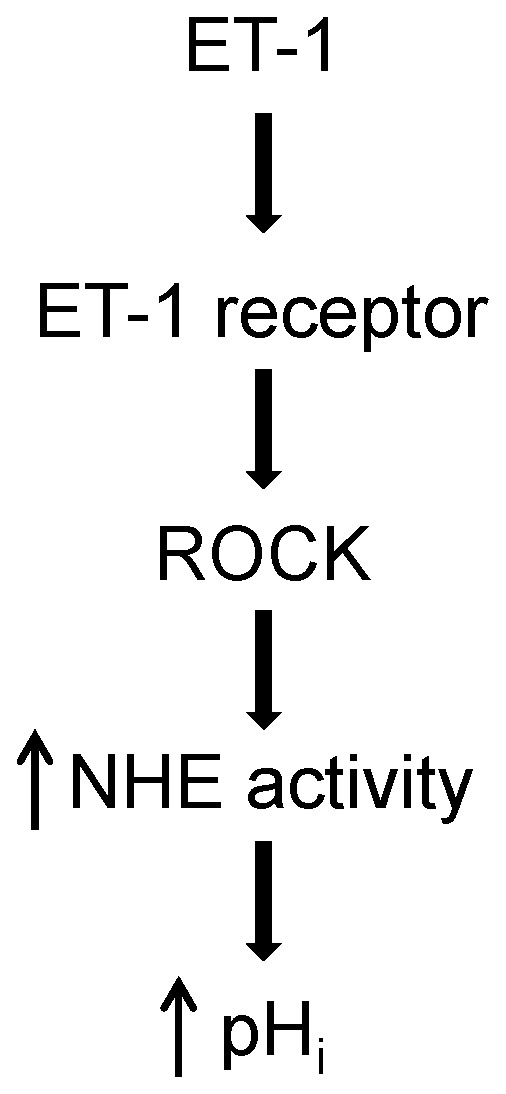
Schematic illustrating the effect of endothelin-1 (ET-1) on intracellular pH (pH_i_) in pulmonary arterial smooth muscle cells. ET-1 binds to surface receptors, leading to activation of Rho kinase (ROCK). ROCK activation in turn results in enhanced Na^+^/H^+^ exchanger (NHE) activity, increased H^+^ efflux and an alkaline shift in pH_i_.

In this study, we demonstrated that acute exposure to ET-1 increased NHE activity in PASMCs, leading to a rapid alkaline shift in pH_i_. The enhancement of NHE activity in response to ET-1 was dependent on ROCK activation, but did not appear to involve activation of PKC (Fig 7). In contrast to previous results reported in systemic vascular smooth muscle [39]–[41], [49], [50], activation of PKC did not stimulate NHE activity or increase pH_i_ in PASMCs.

Our results confirm previous observations [10], [11], [13] that NHE contributes to the regulation of resting pH_i_ in PASMCs, although our data suggest that NHE activity is low under basal conditions, since DMA caused only a small reduction (<0.1 unit) in resting pH_i_ levels. DMA reduced, but did not completely eliminate, basal NHE activity, perhaps due to incomplete inhibition; it is possible that higher concentrations of DMA could have resulted in complete inhibition of NHE activity and a greater reduction in basal pH_i_. Nonetheless, our data is consistent with previously reported results demonstrating a reduction in pH_i_ with DMA or EIPA [11], [13]. Moreover, we found that neither PKC nor ROCK inhibition altered resting pH_i_ or NHE activity, indicating a lack of phosphorylation-dependent activation of NHE in PASMCs under control conditions.

Application of ET-1 increased both pH_i_ and NHE activity in a concentration-dependent manner. The ET-1-induced increase in pH_i_ that we observed in PASMCs is in line with the alkalinizing effect of ET-1 on basal pH_i_ reported in cultured systemic vascular smooth muscle cells [32], [51], [52]. Since the current study was focused on delineating the effects of ET-1 on NHE activity, experiments were performed in CO_2_- and bicarbonate-free solutions, eliminating the effects of the Cl^−/^HCO_3_^-^ exchangers. Determining whether the activity of these exchangers is also altered in the presence of ET-1 or contributes to either enhanced or diminished alkalinization in response to ET-1 will require further investigation.

In previous studies, the modulation of pH_i_ by ET-1 was inhibited by EIPA and Stauro [32], [51], indicating that ET-1 increased the activity of the Na^+^/H^+^ exchanger through a PKC-dependent mechanism. Since PKC activation is a well-recognized consequence of ET receptor activation [53]–[56], and given previous reports supporting PKC-dependent enhancement of NHE activity [38]–[41], we hypothesized that the effects of ET-1 in PASMCs would also be prevented or reduced with PKC inhibition. However, somewhat surprisingly, two structurally unrelated PKC inhibitors failed to block the effects of ET-1 on pH_i_ and NHE activity in PASMCs. Although it might be argued that the lack of effect of GFX could be attributed to a role for non-Ca^2+^-dependent isoforms of PKC [47], Stauro is not selective for specific isoforms of PKC and was also unable to prevent or reduce the effects of ET-1, further confirming that PKC activation is not required for stimulation of NHE activity by ET-1. It is unlikely that the lack of effect of these inhibitors was due to insufficient inhibition of PKC activity, as we have previously shown that these concentrations applied for the same duration of time attenuated the effect of ET-1 on K_V_ currents [48], intracellular Ca^2+^ mobilization [37] and contraction [45], [46] in pulmonary vascular smooth muscle. Experiments in which PKC was directly activated in PASMCs further ruled out a role for PKC, as PMA altered neither pH_i_ nor Na^+^-dependent recovery from acid-loading. These results clearly demonstrate that unlike vascular smooth muscle from systemic sources, PKC activation does not lead to enhanced NHE activity in PASMCs. The reason why is unclear, but it is unlikely that PMA did not activate PKC since the concentrations of PMA used in this study were 2-fold greater than those we previously found activated PKC-dependent Ca^2+^ influx and reduced K_V_ currents in PASMCs within a similar time-frame [37], [48].

Since PKC activation did not appear to mediate the effects of ET-1 on NHE activity in PASMCs, we next focused on the role of ROCK. Binding of ET-1 to either ET_A_ or ET_B_ receptors leads to activation of ROCK in a variety of cell types [55], [57], including PASMCs [58], and ROCK has been implicated as a possible regulator of NHE activity [59]–[61]. We found that inhibiting ROCK prevented both the ET-1-induced increase in NHE activity and the associated alkaline shift in pH_i_, indicating that ROCK activation by ET-1 acutely enhanced NHE activity in PASMCs, most likely due to phosphorylation of one or more known phosphorylation-sensitive sites in the cytoplasmic C-terminal tail region.

It should be noted that the current experiments were performed in PASMCs isolated from normoxic animals and studied under normoxic conditions. In PASMCs isolated from chronically hypoxic rats, we have shown that ET-1-induced activation of ROCK was downstream of PKC activation [37]. However, this does not appear to be the case in PASMCs from normoxic animals since inhibiting PKC and ROCK differentially blocked the effects of ET-1 on pH_i_ and NHE activity. While these results suggest that there may be alterations in ET-1 signaling pathways during chronic hypoxia, whether this is due to activation of different receptors (i.e., ET_A_ versus ET_B_), or indicates that the receptors are coupled to different downstream signaling mechanisms, remains to be determined. Future experiments are also needed to establish whether ET-1 still exerts an acute stimulatory effect on NHE activity in cells from chronically hypoxic animals and, if so, delineate the signaling pathways involved.

It is well-recognized that ET-1 can play a role in the pathogenesis of pulmonary hypertension. In addition to its vasoconstrictive properties, ET-1 also stimulates DNA synthesis and proliferation in PASMCs [33], [62], [63]. Investigation in mice [64] and rats [65] showed that hypoxia induces elevated levels of preproendothelin mRNA as well as ET-1 mRNA in pulmonary tissue. In addition, mRNA levels of ET receptors are elevated in pulmonary tissue after 48 h at 10% O_2_ [66], [67]. The active role of ET-1 in the development of pulmonary hypertension is further supported by studies that have shown inhibition and even reversal of pulmonary hypertension in chronically hypoxic animals treated with ET_A_-receptor antagonists [23], [25], [27], [28], [68], [69]. Clinical studies of pulmonary hypertensive patients also describe elevated plasma levels of ET-1 when compared to normotensive individuals and show higher ET-1 levels in the arterial circuit when compared to the venous circuit pointing to the possibility of a pulmonary origin of ET-1 [70]. Our current data further confirms a role for ET-1 in modulating NHE activity in PASMCs and provides an additional link between ET-1 and the development of pulmonary hypertension.

In summary, we demonstrated that acute challenge with exogenous ET-1 enhances NHE activity in PASMCs via a mechanism involving ROCK activation. Unlike other cell types, PKC activation does not appear to be involved in the regulation of NHE activity and pH_i_ in PASMCs. Since ET-1 can induce PASMC proliferation and enhanced NHE activity has been shown to play a central role in cell growth responses, our results may provide additional insight into the mechanism by which ET-1 modulates PASMCs growth under both physiological and pathologic conditions.
